# Circulating Lymphocyte Subsets Induce Secondary Infection in Acute Pancreatitis

**DOI:** 10.3389/fcimb.2020.00128

**Published:** 2020-03-31

**Authors:** Lili Ding, Yimin Yang, Hongxiang Li, Haijiao Wang, Pujun Gao

**Affiliations:** ^1^Department of Intensive Care Unit, The First Hospital of Jilin University, Changchun, China; ^2^Department of Gynecology Oncology, The First Hospital of Jilin University, Changchun, China; ^3^Department of Hepatology, The First Hospital of Jilin University, Jilin University, Changchun, China

**Keywords:** acute pancreatitis, lymphocyte, lymphopenia, immunosuppression, secondary infection

## Abstract

Acute pancreatitis (AP) is considered a cascade of immune responses triggered by acinar cell necrosis. AP involves two main processes of systemic inflammatory response syndrome and subsequent compensatory anti-inflammatory response syndrome. Although great efforts have been made regarding AP therapy, the mortality rate of AP remains high. Secondary infection acts a lethal factor in AP. Lymphocytes act as major immune mediators in immune responses in the course of this disease. However, the relationship between lymphocytes and secondary infection in AP is unclear. This review summarizes the variation of lymphocytes and infection in AP. Knowledge of the characterization of circulating lymphocyte abnormalities is relevant for understanding the pathophysiology of AP.

## Introduction

Acute pancreatitis (AP) is an inflammatory disease that is commonly caused by either genetic or acquired reasons and is accompanied by a complex cascade of immunological events that affect its pathogenesis and progression (Mayerle et al., 2012). Gallstones, hypertriglyceridemia, and alcohol abuse are the major etiological factors of AP, and these contribute to ~80% of cases (Van Acker et al., 2007). Abnormally activated trypsinogenin *in vivo* causes damage to the pancreatic acini and auto-digestion of the pancreas. Proinflammatory cytokines or chemicals are released into the circulation and enhance the permeability of capillaries of organs. Consequently, pancreatic edema, hemorrhage, and multiple organ dysfunction syndrome occur (Shamoon et al., 2016). AP presents as either mild AP (MAP) or severe AP (SAP) without/with systemic complications or organ dysfunction. SAP is a serious pancreatic inflammatory disease that follows two major courses: systemic inflammatory response syndrome (SIRS) and compensatory anti-inflammatory response syndrome (CARS). SIRS is characterized by a cascade of inflammatory mediators. CARS results from immunosuppression and leads to sepsis-related complications. Although great progress has been made in AP therapy in recent decades, its mortality rate can reach up to 30% in severe cases (Eland et al., 2000; Gravante et al., 2009). Indeed, 66–80% of late mortality in SAP contributes to septic complications (Thomson et al., 2019).

Immune responses are involved in the pathogenesis of SAP. Cellular immunity plays a crucial role in the immune system. Multiple types of cells, including neutrophils, mast cells, monocytes/macrophages, and dendritic cells, are involved in progression of AP (Pietruczuk et al., 2006; Fonteh et al., 2018). Besides innate immune cells, adaptive immune cells also play an important role in the development of AP. Abnormal activation of T and B lymphocytes is thought to be an important factor in modulation of the inflammatory reaction in AP (Mora et al., 1997). The count and function of lymphocytes vary in AP compared with the healthy population and secondary infection also commonly occurs during the disease course. However, the relationship between lymphocytes and secondary infection in AP has rarely been discussed. In this review, we summarize the variation of lymphocytes and their effect on secondary infection in AP.

## Lymphopenia in AP

The pancreas is a major source of proinflammatory cytokines in AP because pancreatic acinar cells damaged by trypsinization are considered as the initiative factor during the early stage of AP. Early expression of genes for interleukin-1 (IL-1) and tumor necrosis factor-α (TNF-α) commonly occurs in AP. In humans, serum IL-1, IL-6, and TNF-α are correlated with the severity of illness (Sweeney et al., 2003). Cytokines such as TNF-α, IL-6, IL-10, and chemokine monocyte chemoattractant protein-1 (MCP-1; CCL2), which are released by damaged acinar cells, recruit leukocytes to injured pancreatic acinar cells and trigger a series of immune responses (Gukovskaya et al., 1997; Gu et al., 2013). The number of leukocytes in AP is significantly higher, but total circulating lymphocytes (T cells, B cells, natural killer [NK] cells) are markedly lower than those in healthy controls, especially in severe cases (Pezzilli et al., 1995; Ueda et al., 2006; Dabrowski et al., 2008; Wei et al., 2019). The characteristics of immune responses triggered by circulating lymphocytes vary in different phases of AP. In the early stage of AP, the activation markers CD69, CD25, CD28, CD38, and CD122 are increased on the lymphocyte surface, which suggests the activated status of T, B, and NK cells (Takeyama et al., 2000; Pezzilli et al., 2003; Sweeney et al., 2003). Activation of lymphocytes results in high levels of proinflammatory cytokines, such as IL-6 and TNF-α. However, abnormal T cell activation has been reported only in MAP, but not in severe cases (Sweeney et al., 2003). In the late phase of AP, the count of peripheral blood lymphocytes recovers, except for in patients with infection. However, studies on AP have reported that the number of B lymphocytes decreases more persistently than T lymphocytes, even at day 30 (Pietruczuk et al., 2006; Zhang X. P. et al., 2009). Consequently, humoral and cellular immune responses are inhibited at different levels.

Absolute lymphocytes in patients with AP have been reported and the subsets of these cells have also been described. Several studies have shown a reduced number of circulating peripheral CD4^+^ T cells, and these subsets subsequently increase within 5 days. Moreover, a decrease in CD4^+^ T cells indicates a serious illness and poor prognosis *in vivo* (Pietruczuk et al., 2006; Ueda et al., 2006; Liu et al., 2015; Yang et al., 2015). CD8^+^ T cells, B cells, and NK cells are also involved in AP. However, the levels of CD8^+^ T cells are still controversial in AP because there is no consistent trend in SAP and MAP. Additionally, levels of soluble CD4, soluble CD8, and soluble IL-2 receptor are increased in cases of AP (Uehara et al., 2003). Different from T cells, depletion of NK cells recovers until the late stage of AP, and then they recover to normal levels on the 30th day in progression of this disease (Fonteh et al., 2018).

Takeyama et al. (2000) found that the main reason for depletion of lymphocytes in AP was apoptosis by cell cycle analysis. They showed that the proportion of apoptotic cells was nearly one quarter of total lymphocytes after incubation for 24 h. Studies have indicated that reduced absolute lymphocytes may be associated with apoptosis via Fas/FasL signaling in CD8^+^ T cells (Qin et al., 2013; Pinhu et al., 2014). These findings strengthen the hypothesis of subsequent CARS in AP (Pinhu et al., 2014). Additionally, depletion of circulating lymphocytes is due to migration of activated lymphocytes to the inflammatory site *in vivo*, such as the pancreas and lungs (Bhatia et al., 2005). Consequently, humoral and cellular immune responses are both inhibited to different degrees and secondary infection is likely to occur in patients with AP (Pietruczuk et al., 2006).

### T Cells

Naïve CD4^+^ T cells can differentiate into various T helper (Th) cell subsets in a certain environment by their pattern of cytokines and transcription factors as follows: Th1, Th2, Th9, Th17, and Th22 cells, follicular helper T cells (TFH), and regulatory T (Treg) cells. Treg cells are divided into two subsets according to different origins of inducible Treg cells and natural Treg cells.

#### Th1 and Th2 Cells

Th1 cells are characterized by expression of interferon (IFN)-γ. Th2 cells play a crucial role in resisting infection by parasites. The symbolic cytokines of Th2 cells are IL-4, IL-5, and IL-13, which play a role in multiple immune cells (such as eosinophils, basophils, mast cells, and B cells; Zhu and Paul, 2008). Rodriguez-Nicolas et al. (2018) found that serum IFN-γ, IL-6, and TNF-α levels were high in SAP, whereas granulocyte-macrophage colony-stimulating factor, IL-4, IL1-β, and IL-13 levels were high in mild/moderately severe AP. Additionally, Serum IFN-γ, TNF-α, IL-5, IL-6, and IL-10 levels were significantly higher in patients with SAP or moderately SAP than in those with MAP. These findings suggest that the Th1 profile is associated with SAP, while the Th2 profile is associated with MAP or moderately SAP. Indeed, the frequency of Th1 is remarkably decreased in the initial days of SAP, but gradually decreases and even reverses over time. Conversely, the number of Th2 cells is increased in the early stage of SAP, but it decreases over the course of SAP. A dynamic balance in Th1/Th2 cells is present in patients with SAP, especially in the early stage, which indicates immunopathogenic changes in patients with SAP (Li et al., 2013).

#### Th9 Cells

Th9 preferentially produces IL-9, contributing to effective immunity and immunopathology in multiple diseases, including autoimmune diseases, tumors, and infectious diseases (Kaplan et al., 2015). Moreover, IL-9^+^ T cells commonly lack IL-13 and IFN-γ *ex vivo*, and IL-9^+^ T cells can increase numbers of infiltrating eosinophils, basophils, and mast cells (Jones et al., 2012). Furthermore, AP is characterized by sterilized inflammation of the pancreas, which involves eosinophils, basophils, mast cells, and innate immunity. The finding of Th9 cells initiating immunity to helminth parasites indicates the possibility of these cells regulating immune responses in AP (Licona-Limón et al., 2013). In edematous and necrotizing porcine pancreatitis models, IL-9 levels in blood do not change compared with the sham group in the early phase (Merilainen et al., 2012). However, there have not been any reports on the number or function of Th9 cells involved in initiation or progression AP.

#### Th17 Cells

Th17 cells differentiate from naïve CD4^+^ T cells and they play a role in protecting the body against ectogenic pathogens. The presence of transforming growth factor (TGF)-β and IL-6 facilitates Th17 differentiation from naïve CD4^+^ T cells in mice (Binger et al., 2017). The signature cytokines of Th17 cells are IL-17A, IL-17F, and IL-22 (Park et al., 2005; Veldhoen et al., 2006). IL-17 is a proinflammatory cytokine that controls extracellular pathogens and induction of matrix destruction. Th17 cells are considered as an important regulator in multiple diseases, such as multiple sclerosis, psoriasis, and inflammatory bowel disease (Miossec et al., 2009). IL-17 is secreted by Th17 cells, NK cells, and natural killer T (NKT) cells. Furthermore, high IL-17 levels from T cells or other subsets are associated with organ or tissue chronic inflammation. In AP, IL-17 targets innate immune cells and epithelial cells to produce granulocyte colony-stimulating factor and IL-8, which facilitate the production and recruitment of neutrophils (Weaver et al., 2013). These neutrophils are activated and release high concentrations of oxidants and cytotoxic agents, which mediate a cascade of immune responses and remote organ dysfunction. Circulating IL-6 and IL-10 levels are elevated in patients with AP (Mentula et al., 2005). These cytokines may contribute to constitutive expression of pSTAT3, which favors Th17 differentiation from CD4^+^ lymphocytes (Egwuagu, 2009). This cytokine–pSTAT3–cell cycle may enhance progression of AP. In fact, serum IL-17 levels are elevated and correlated with the severity of AP. IL-17 serves as a potential prognostic marker for patients with SAP (Botoi and Andercou, 2009; Jia et al., 2015). Furthermore, a previous study showed that the IL-17^+^ cell percentage in circulation of patients with SAP was significantly related to disease severity (Wang et al., 2018). This finding indicates that elevated numbers of Th17 cells lead to deterioration of this disease. A recent study showed that IL-17A^+^ cells were increased in human chronic pancreatitis tissues (Zhao Q. et al., 2019). This study also showed that decreased IL-17A^+^ cells in the pancreas alleviates chronic pancreatitis-associated inflammation and fibrosis via stimulation of interferon gene signaling, which suggests that Th17 is involved in chronic inflammation of the pancreas.

#### Th22 Cells

Th22 cells mainly secrete cytokines, including IL-22, IL-13, and TNF-α. Th22 cells are CD4^+^ effector T cells, and the transcription factor aryl hydrocarbon receptor is crucial for generation of Th22. Th22 cells are commonly present in peripheral blood or the epidermal layer in inflammatory skin (Duhen et al., 2009). Th22 cells are involved in multiple diseases, including inflammatory diseases, autoimmune diseases, and tumors (Jia and Wu, 2014). Th22 cells often play a dual role of promotion and protection in diseases. IL-22, which is the signature cytokine of Th22, is elevated in plasma in patients with AP (Vasseur et al., 2014). A study showed that recombinant IL-22 ameliorated the severity of cerulein-induced pancreatitis in mice (Feng et al., 2012). However, in lung tissues of experimental rats with AP, mRNA levels of IL-22 and Th22 are lower in rats with SAP than in rats without SAP, and levels of mRNA encoding IL-22 and Th22 are reduced with development of SAP (Huai et al., 2012). Couturier-Maillard et al. found that IL-22 deficiency exacerbated toxoplasma gondii-induced intestinal inflammation in mice (Couturier-Maillard et al., 2018). These findings suggest that IL-22 plays a protective role in either inflammatory or infectious disease. The protective mechanism of IL-22 in AP is inhibition of autophagosome formation via inducing Bcl-2 and Bcl-XL, which bind to Beclin-1 (Feng et al., 2012). Aryl hydrocarbon receptor activation is another protective factor by promoting expression of IL-22. The aryl hydrocarbon receptor regulates the immune response between pancreatic leukocytes and epithelial cells. Consequently, the aryl hydrocarbon receptor is a potential therapeutic target of AP. Notably, these findings mainly demonstrate the protective role of IL-22 instead of Th22. Because IL-22 is not only secreted by Th22, more studies are required to confirm the precise role of IL-22-secreting cells in peripheral blood or inflammatory tissues in AP. At present, there have been no studies on Th22 in patients with AP.

#### TFH Cells

TFH cells are also known as follicular B helper T cells and are located in peripheral secondary lymphoid organs. These cells are characterized by expression of the B cell follicle homing receptor chemokine receptor type 5 (Fazilleau et al., 2009). The main function of TFH cells is to promote and maintain germinal centers via CD40 ligand expression and IL-21 secretion. IL-21 is related to inflammation, autoimmunity, and infection. At mRNA and protein plasma levels, IL-21 is elevated in patients with SAP compared with those with MAP, but the trend is not statistically significant. Moreover, in the late phase of AP, IL-21 levels are elevated in septic patients with SAP and in patients with pancreatic necrosis, which suggests a potential role of IL-21 in AP with necrosis and sepsis (Thomson et al., 2019). Therefore, TFH cells may be involved in progression of AP. However, IL-21 is not only secreted by TFH cells, but is also expressed by Th17 cells, TFH cells, and Th2 cells. At present, the precise role of TFH is still not well-known in AP.

#### Treg Cells

Treg cells inhibit the activity of dendritic cells, NKs, and other immune cells in the pancreas, and inhibit an exaggerated inflammatory response (Kim et al., 2007; Feuerer et al., 2009). Conversely, inhibition of Treg cells results in uncontrolled inflammation because this type of cell inhibits the immunocompetence of macrophages and promotes them to differentiate toward an M2 phenotype (Tiemessen et al., 2007). The Treg cell count in AP is still controversial. Li et al. (2013) showed that the count of Treg cells continuously increased in the disease course. A high percentage of Treg cells is associated with continuous immune suppression. Additionally, a persistently high percentage of Treg cells in peripheral blood is a sign of a secondary activated immune response on the basis of increased secretion of anti-inflammatory cytokines, such as IL-10 and TGF-β (Chen et al., 2015; Zheng et al., 2015). Furthermore, a high percentage of natural Treg cells is associated with an unfavorable outcome (Minkov et al., 2017). However, the percentage of CD4^+^CD25^+^CD127^low/neg^ cells of the total CD4^+^ cells in patients with AP is remarkably lower compared with that in healthy controls (Wang et al., 2017). In a mouse SAP model, investigators reported that the number of CD4^+^CD25^+^ T cells was markedly lower compared with the sham group (Zheng et al., 2015). Feuerer et al. (2009) showed that a reduced number of Treg cells in experimental mice promoted activation of the local CD4^+^ T effector cell population in the pancreas, and this cell population aggressively attacked the islets. This is a potential reason for autoimmune diabetes. The biomarkers of Treg cells are different in different studies, with either CD4^+^CD25^+^CD127^−/neg^ or CD4^+^CD25^+^Foxp3^+^ contributing to different Treg cell counts or trends in different studies. Additionally, a shortage of simultaneous healthy control data contributes to variation of Treg cells. At present, data on Treg cells in humans with AP are still limited and the precise function of Treg cells in AP requires further study. TGF-β has the characteristic of controlling immune responses and maintaining immune homeostasis. TGF-β is also involved in process of Th17 and Treg differentiation, which suggests a close relationship between Th17 and Treg cells (Korn et al., 2009). In autoimmune diseases, TGF-β regulates the Th17/Treg ratio via specific signaling, such as T cell receptor signaling, costimulatory signals, and cytokine signaling (Lee, 2018). However, precise regulation of TGF-β in the Th17/Treg ratio in AP is still not well known. A previous study showed that the Th17/Treg ratio was correlated with severe AP, and the balance of this ratio was regulated by mi-RNA 155 (Wang et al., 2018).

#### CD8^+^ T Cells

CD8^+^ T cells are regarded as cytotoxic T cells in the immune system and contribute to the adaptive immune response. This response kills cells infected by intracellular bacteria, intracellular viruses, and cancer cells via releasing perforin and granulysin or cell-surface interactions. Absolute circulating number of cytotoxic T cells is decreased in SAP (Dabrowski et al., 2008). A reduced number of cytotoxic T cells is associated with overexpression of Fas/FasL, which induces apoptosis of lymphocytes in AP (Pinhu et al., 2014). Furthermore, a decreased number of cytotoxic T cells is associated with infection in AP, which indicates that cytotoxic T cells play a protective role in AP. Notably, CD8^+^ suppressor cells are a subset of CD8^+^ T cells, which have immunosuppressive functions by inhibiting antibody synthesis or cell-mediated immunity (Churlaud et al., 2015). CD8+ suppressor cells are involved in autoimmune diseases, including inflammatory bowel disease and systemic lupus erythematosus (Brimnes et al., 2005; Zhang L. et al., 2009). CD8^+^ suppressor cells are believed to play an active role in mucosal tolerance. However, there have not been any studies regarding CD8^+^ suppressor cells in AP. CD8^+^ Tregs are another subpopulation of CD8^+^ T cells with an inhibitory effect. CD8^+^ Tregs play an immunosuppressive role by secretion of IL-10 and TGF-β or by activating negative signaling in a cell–cell contact manner (Vieyra-Lobato et al., 2018). CD8^+^ Tregs are involved in various diseases, such as autoimmune diseases, tumors, and graft-versus-host disease. There have been no reports concerning the relationship between CD8^+^ Tregs and pancreatitis. Notably, the number of CD8^+^ Tregs is positively correlated with survival in patients with adenocarcinoma of the pancreas (Davis et al., 2012). Furthermore, a study that focused on a pancreatic graft from a patient with type 1 diabetes who had normal endocrine function after kidney–pancreas transplantation showed an increase of CD8^+^ Tregs in the pancreas (Velthuis et al., 2009). These studies indicate that CD8^+^ Tregs may play a role in pancreatic diseases, including AP.

### B Cells

B cells are a subset of lymphocytes and the primary function of B cells is to mediate humoral immunity in the adaptive immune system by secreting antibodies. The number of B lymphocytes (CD19^+^) is significantly reduced either in the early or late stage of SAP, and this state persists for longer in SAP than in MAP (Pietruczuk et al., 2006). When these patients become infected, the decreased percentage of B lymphocytes is more remarkable. However, the number of B lymphocytes is at a high level in non-infected patients (Shen and Cui, 2012). B cells in SAP and hyperlipidemia-AP are significantly elevated and the number of B cells is positively related to the length of hospital stay in patients with MAP (Wei et al., 2019). The B cell subset in the spleen and liver in the necrotizing AP model is remarkably reduced (Schmidt et al., 2017). A recent study showed a reduced frequency of CD4^−^CD8^−^ double negative T cells in patients with AP (Wei et al., 2019). A previous study also showed that *ex vivo* converted CD4^−^CD8^−^ T cells suppressed B cell proliferation and induced apoptosis of B cells by perforin or in a cell–cell contact-dependent manner (Li et al., 2014). Consequently, the effective factor of B cells (immunoglobulin) varies in AP compared with healthy controls. A previous study showed that immunoglobulin M (IgM) levels in serum and anti-endotoxin antibody IgG levels were below the normal range in SAP (Sharma et al., 2012). Moreover, another study showed that IgG and IgM levels in serum were below the normal range in infectious patients with AP and IgG levels were lower in non-survivors compared with survivors, but serum IgA levels were unchanged (Ueda et al., 2006). Suppression of B cell function suggests chronic and increasing impairment of the humoral immune response. Regulatory B cells are the main subtype of B cells and they play an immunosuppressive function that prevents expansion of proinflammatory cells by secretion of IL-10, IL-35, and TGF-β (Rosser and Mauri, 2015). The two main B subsets are memory CD19^+^CD24^hi^CD27^hi^ (Iwata et al., 2011) and immature/transitional CD19^+^CD24^hi^CD38^hi^ B cells (Oleinika et al., 2019), both of which can secrete IL-10 in certain stimulations. CD19^+^CD24^hi^CD38^hi^ cells inhibit Th1 and Th17 cell differentiation by secretion of IL-10 (Flores-Borja et al., 2013). A decrease in circulating IL-10-producing B cells and CD19^+^CD24^hi^CD27^hi^ cells has been observed in patients with AP, and this trend is more obvious in those with SAP than in those with MAP (Qiu et al., 2018). These two subsets are negatively correlated with the severity of AP. In MAP, these two subsets of cells are significantly increased from days 1 to 7 (Qiu et al., 2018), which indicates that the severity of disease is alleviated accompanied by elevation of regulatory B cells. Regulatory B cells may act as predictors in development of SAP.

### NK Cells

NK cells are a subtype of lymphocytes by their properties of cytolytic lymphocytes, which are distinct from B and T lymphocytes. NK cells play an important role in innate immunity and adaptive immunity (Vivier et al., 2011). The primary function of NK cells is to kill virus-infected cells and tumor cells by perforin and granzyme (Orr and Lanier, 2010). A reduced number of NK cells in peripheral blood in AP was shown decades previously. Additionally, a reduced range of this subset is more remarkable in SAP than in MAP (Dabrowski et al., 2008; Wei et al., 2019). The most dramatic depletion of lymphocytes in the circulation is CD3^−^CD16^+^56^+^ cells in the first 24 h of AP. This suggests that the frequency of NK cells in the circulation is a potential biomarker for prognosis of AP (Zhao Z. et al., 2019).

### NKT Cells

NKT cells are a heterogeneous subtype of T cells. NKT cells are characterized by the properties of T cells and NK cells, such as CD16 and CD56 expression and granzyme production (Vivier and Anfossi, 2004). NKT cells account for ~0.1% of total T cells in the circulation. A previous study on innate immune responses to an adenoviral vector showed that NK and NKT cells migrated to the pancreas and persisted in the pancreas after vector administration (Shifrin et al., 2005). This finding indicates that NK and NKT cells play a main role in the progress of acute adenovirus-mediated pancreatitis.

## Lymphopenia and Infection

A reduced number of lymphocytes is called lymphopenia. Lymphopenia is a pathological state in which the peripheral lymphocyte count is below 1.0 to 1.5 × 10^9^/L (Saroha et al., 2013). A prospective cohort study involved 98,344 individuals from the general population showed that lymphopenia was an independent risk factor with infection and hospitalization (Warny et al., 2018). Moreover, the risk of death associated with infection has increased by 1.7 times in individuals with lymphopenia (Warny et al., 2018). Another study of 753 patients in the intensive care unit showed that lymphopenia increased the infectious risk as high as 1.6-fold (Adrie et al., 2017). Notably, in this study, persistent lymphopenia increased the risk of 28-days mortality to 1.7 times. Additionally, this increased risk of infection or mortality is more obvious in autoimmune or malignant diseases (Merayo-Chalico et al., 2013; Saroha et al., 2013). In conclusion, lymphopenia reduces the ability of the body to resist infection. Patients with AP, especially those with SAP, tend to develop infection compared with healthy controls. However, studies concerning lymphopenia and AP are limited at present.

## Individuals With AP Are Likely to Develop Infection

Infection occurs in ~20% of patients with AP. However, this ratio is as high as 40% if patients have SAP. The gut protects organisms from sepsis, cytokine-mediated SIRS, multiple organ dysfunction syndrome, and even death by pathogenic bacteria and their antigens in the gut (Penalva et al., 2004; Liu et al., 2008). Some natural and adaptive immune cells colonize non-lymphoid tissues, which plays a role in resistance to pathogens, homeostasis, and immune regulation. These cells do not participate in the blood or lymph circulation and have a unique phenotype in tissue that is different from immune cells in the circulation. In AP, the extrapancreatic infection rate is ~24% in the early stage of patients with AP, and this infection is related to a doubling in mortality rate (Wu et al., 2008; Besselink et al., 2009). Secondary infections are mostly caused by intestinal flora penetrating the abdomen. This is mainly due to a disorder of gastrointestinal function.

Researchers have found that endotoxin, the lactulose/mannitol ratio, and D(-)-lactate levels, which reflect intestinal permeability and gut barrier dysfunction, continuously decrease during the first 2 weeks of SAP (Li et al., 2013). Intestinal permeability is higher in patients with AP compared with healthy controls. This high permeability is also more persistent in SAP than in MAP (Schietroma et al., 2016). Additionally, endotoxin levels are related to intestinal permeability (Penalva et al., 2004). Therefore, endotoxemia in AP may result from hyperintestinal permeability. Furthermore, gastrointestinal extension, which is present as abdominal compartment syndrome (ACS), facilitates microbiota from the intestine to translocate to the abdominal cavity. Intra-abdominal hypertension is defined as intra-abdominal pressure more than 12 mmHg and ACS. Researchers have shown that 38% of patients with acute pancreatitis develop ACS (van Brunschot et al., 2014). Bacterial ectopic colonization and its associated inflammatory complications may be due to intestinal flora or its products entering the bloodstream through a dysfunctional intestinal barrier (Sharif et al., 2009). Additionally, cytokines and chemokines in the bloodstream can increase capillary permeability by inducing secretion of acute proteins and activating complement and bradykinin–kinin systems. This increased capillary permeability, including the gut, facilitates bacterial translocation into the systemic circulation and causes sepsis (Bossi et al., 2011).

## Infectious Events in AP

CARS commonly occurs in the late stage of AP. CARS is characterized as immunosuppression, which is mostly likely mediated by secondary infection. Lymphopenia is present in most patients with AP, especially in SAP. Interestingly, most infection occurs in SAP instead of MAP. Lymphopenia appears to contribute to CARS/immunosuppression and facilitates development of infection in AP. In fact, a study showed that depletion of immune cells in AP and inhibition of immunity increased secondary infection, especially in SAP cases (Shen et al., 2015). Furthermore, a continuous immunosuppressive status results in a high risk of infection. More than 80% of mortality that occurs in the late phase in AP results from infection (Gloor et al., 2001). Investigators who focused on the subsets of immune cells found that an imbalance in the CD4^+^/CD8^+^ ratio was attributed to secondary infection in AP (Shen and Cui, 2012). The CD4^+^/CD8^+^ ratio was higher in patients with secondary infection compared with non-infected cases during the first 7 days, but the CD4^+^/CD8^+^ ratio was markedly decreased by 28 days. Liu et al. (2015) showed that ACS could be predicted by reduced circulating CD4^+^ T lymphocytes and the CD4^+^/CD8^+^ ratio in patients with SAP. ACS results from high penetration of the intestine and facilitates bacteria to translate to the abdomen and causes early death in patients with SAP. A decreased frequency of CD8^+^ T lymphocytes is associated with secondary infection in the early phase of pancreatitis (Takeyama et al., 2000). Additionally, patients with AP have low serum IgM levels, and serum IgG levels are below the normal range in patients with infection in AP. Furthermore, low lymphokine-activated killer cell activity, NK cell activity, and antibody-dependent cellular cytotoxicity activity are present in patients with AP (Ueda et al., 2006). Expression of IFN-γ is inhibited in AP, which aggravates the severity of this disease. Rau et al. (2006) found that treatment with recombinant rat IFN-γ in experimental rats with SAP postponed necrosis of the pancreas, reduced infiltration of neutrophils, and decreased IL-1 secretion in the pancreas. These findings indicate that decreased levels of Thl cytokines, such as IL-2, IL-12, and IFN-γ, increase immunosuppression in SAP (Zhang X. P. et al., 2009). In conclusion, depletion of lymphocytes and the related cytokine variation maintain immunosuppression in AP, which facilitates a second wave of infection.

## Summary

**Figure 1:**
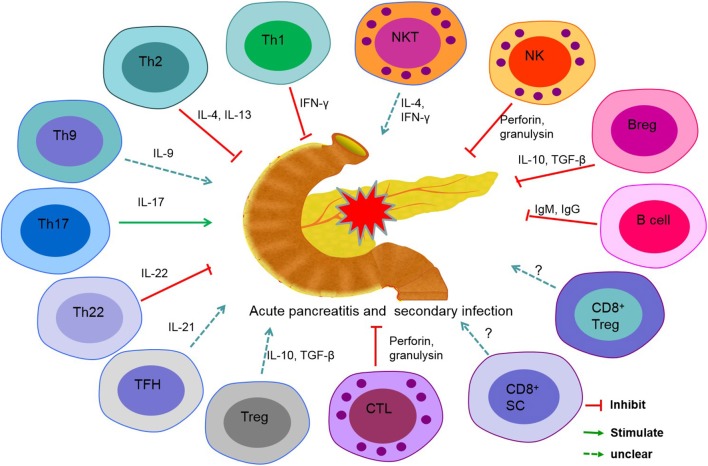
Role of lymphocyte subsets in secondary infections in acute pancreatitis (AP). T helper (Th) cell 1, Th2, Th22, cytotoxic T lymphocyte (CTL), B cell, regulatory B (Breg) cell, and natural killer (NK) cell play a protective role in secondary infection in acute pancreatitis. Th17 and interleukin-17 (IL-17) promote inflammation of the pancreas and induce secondary infection. The precise roles of Th9, follicular helper T (TFH) cell, regulatory T (Treg) cell, CD8+ suppressor cell (CD8^+^ SC), CD8^+^ Treg, and natural killer T (NKT) cell are unclear in AP.

AP is considered the result of excessive inflammation in the pancreas or in multiple organs. A lethal factor of this disease is secondary infection, which usually occurs in the CARS phase of AP. In patients with AP, lymphopenia mainly contributes to immunosuppression and facilitates bacterial invasion and growth. Additionally, ACS, certain cytokines, and chemokines play accessory roles in the course of AP. Several subsets of lymphocytes have direct or indirect effects on the prognosis of AP (Figure 1). However, newly discovered lymphocytes, such as Th22 cells, innate lymphoid cells, and mucosal-associated invariant cells, which play an essential role in inflammatory diseases, autoimmune diseases, or tumors, have not been reported in patients with AP. Revealing the mechanisms of immunosuppression in AP may lead to a better prognosis of AP.

## Author Contributions

LD, YY, HL, and HW wrote this review. PG revised the manuscript.

### Conflict of Interest

The authors declare that the research was conducted in the absence of any commercial or financial relationships that could be construed as a potential conflict of interest.
